# A Plea for a New Synthesis: From Twentieth-Century Paleobiology to Twenty-First-Century Paleontology and Back Again

**DOI:** 10.3390/biology11081120

**Published:** 2022-07-26

**Authors:** Marco Tamborini

**Affiliations:** Department of Philosophy, Technische Universität Darmstadt, Marktplatz 15 (Residenzschloss), 64283 Darmstadt, Germany; marco.tamborini@tu-darmstadt.de

**Keywords:** paleontology, paleobiology, history and philosophy of paleontology, twenty-first-century paleontology, paleobiological revolution, technoscience and global issues

## Abstract

**Simple Summary:**

This article examines the relationship between twentieth- and twenty-first-century paleobiology. After summarizing the disciplinary problem of paleontology in the mid-twentieth century, I focus on five representative research topics in contemporary paleontology. In doing so, I outline twenty-first-century paleontology as a science that seeks more data, more technology, and more integration. At the end of the paper, I highlight a possible new paleobiological revolution: it would give paleontologists strong political representation to deal with issues such as expanded evolutionary synthesis, conservation of the Earth’s environment, and global climate change.

**Abstract:**

In this paper, I will briefly discuss the elements of novelty and continuity between twentieth-century paleobiology and twenty-first-century paleontology. First, I will outline the heated debate over the disciplinary status of paleontology in the mid-twentieth century. Second, I will analyze the main theoretical issue behind this debate by considering two prominent case studies within the broader paleobiology agenda. Third, I will turn to twenty-first century paleontology and address five representative research topics. In doing so, I will characterize twenty-first century paleontology as a science that strives for more data, more technology, and more integration. Finally, I will outline what twenty-first-century paleontology might inherit from twentieth-century paleobiology: the pursuit of and plea for a new synthesis that could lead to a second paleobiological revolution. Following in the footsteps of the paleobiological revolution of the 1960s and 1970s, the paleobiological revolution of the twenty-first century would enable paleontologists to gain strong political representation and argue with a decisive voice at the “high table” on issues such as the expanded evolutionary synthesis, the conservation of Earth’s environment, and global climate change.

## 1. Introduction

At the beginning of his book *Tempo and Mode in Evolution* (1944), North American paleontologist George Gaylord Simpson (1902–1984) provided an interesting picture of the difficulties and lack of interaction between the paleontological and biological communities during the middle of the twentieth century. He wrote, “not long ago […] geneticists said that paleontology had no further contributions to make to biology, that […] it was a subject too purely descriptive to merit the name “science”. The paleontologist, they believed, is like a man who undertakes to study the principles of the internal combustion engine by standing on a street corner and watching the motor cars whiz by” [1].

The same opinion was shared by prominent biologists. For instance, British geneticist John Maynard Smith (1920–2004) noted that that during the 1960s there were continuous frictions between paleontologists and biologists. As he recalled in 1984, “[at] that time, the attitude of populations genetics to any paleontologist rash enough to offer a contribution to evolutionary theory has been to tell him go away and find another fossil, and not to bother the grownups” [2]. However, in the same piece, Maynard Smith added two lines, in effect showing the different perception and value of paleontology. He admitted, “Palaeontology has been too long absent from the high table. Welcome back” [2].

In welcoming paleontology to the table that matters, the geneticist paved the way for some historical and theoretical questions: What happened between the 1960s and 1980s that was so imprinted as to make his judgment change? Does twenty-first-century paleontology still sit at the high-table? And how does the scientific agenda of today’s paleontology relate to its scientific past and future?

In this paper, I will briefly address these questions, in effect reflecting on the elements of novelty and continuity between twentieth-century paleobiology and twenty-first-century paleontology. First, I will expose the heated debate about the disciplinary status of paleontology during the mid-twentieth century. Second, I will analyze the main theoretical issue behind this debate by looking at two prominent case studies within the broader paleobiological agenda. Third, I will move to twenty-first-century paleontology and address five representative research topics. By doing so, I will characterize twenty-first-century paleontology as a science that seeks *more data, more technology, and more integration*. Moreover, I will I focus on the notion of the “second digital revolution” as it occurs in paleontology. In the conclusion, I will state what twenty-first-century paleontology might take from twentieth-century paleobiology: the aspiration of and plea for a new synthesis that may lead to a second paleobiological revolution.

## 2. Paleobiology vs. Paleontology

“We are paleontologists, so we need a name to contrast ourselves with all you folks who study modern organisms in human or ecological time. You therefore become neontologists” [3]. With his classical provocative and quite “parochialism” jargon, North American paleontologist Stephan Jay Gould (1941–2002) called attention to two important elements that characterized the methodology and disciplinary status of paleontology throughout the twentieth century. First, Gould insisted that a different name was necessary to differentiate two quite different approaches to evolution. According to Gould, the term ‘paleontology’ was too weak and too historically laden to achieve the aim of promoting paleontologists as genuine biologists and not as geologists. Together with several US colleagues, such as Niles Eldredge, David Raup, Jack Sepkoski, Steven Stanley, and others, he chose the term “paleobiology” as the perfect name to describe paleontological interests in evolutionary biology [4,5]. Paleobiology had its heyday between 1970 and 1985. This name though goes back to Austrian paleontologist and biologist Othenio Abel (1875–1946). He coined the term “paleobiology” to emphasize the biological meaning of this discipline. Paleobiology, so Abel, was able to investigate evolutionary mechanisms, in effect providing the deep-time perspective to evolutionary study [6,7,8]. Hence, paleontology should be considered a biological sub-discipline and not practiced in geological departments.

Second, Gould contrasted between evolutionary investigations of living organisms (what he called neontological analyses) and the paleo(bio)ontological ones, which study what happened in the deep past. Indeed, Gould’s statement derived from the empirical results he himself and other paleontologists were obtaining during the 1960s and 1970s. These scientists were keen to present their discipline as a rigorous investigation of evolutionary mechanisms. By uncovering deep-time patterns and processes, paleontologists were indeed able to contribute to and expand the evolutionary mechanisms set during the modern synthesis of evolution. Gould coupled this theoretical aim with a broader idea of science, paleontology, and evolutionary time [5,9,10,11].

As noted, the second ground behind Gould’s provocative statement was his emphasis on the importance of deep-time investigations as defining characteristic of paleontology. Although the discovery and conquest of deep time was a classical argument for the importance of paleontology since the seventeenth century [12,13,14,15,16], this dimension acquired extra (biological) value in the twentieth century. In 1985, Gould published a paper in the journal *Paleobiology*, arguing that “Nature’s discontinuities occur at different scales of time or tiers”. He distinguished three distinct temporal tiers. The first tier includes events at the ecological movement; the second tier encompasses events which occur during “millions of years in “normal” geological time”; whereas the most exciting subject in paleontology lies in our recognition that one of our best-recognized and most puzzling phenomena, mass extinction, is not merely more and quicker of the same, but a third distinct tier with rules and principles of its own [17].

Successively, he stated that “whatever accumulates at the first tier is sufficiently reversed, undone, or overridden by processes of the higher tiers” [17]. For instance, mass extinction occurs at the third tier. It “works by different rules and may undo whatever the lower tiers had accumulated” [17]. Evolution is an extremely hierarchical phenomenon. Hence, Gould noted that only paleobiologists are able to investigate what happened in the second and third tier; whereas “neontologists” study “modern organisms in human or ecological time” [17].

Gould’s idea was that evolutionary time could be seen as a system of distinct tiers—and the problem of transpacific evolution requires an explicit study of their interaction. Darwinian tradition leads us to deny this kind of structuring, to view time as a continuous, and to seek the source of causality at all scales in observable events and processes at smallest [9,18].

To put it simply, Gould and colleagues meant to “(1) make paleontology more theoretical and less descriptive; (2) introduce models and quantitative analysis into paleontological methodology; (3) import ideas and techniques from other disciplines (especially biology) into paleontology; (4) emphasize the evolutionary implications of the fossil record” [5]. Or, as philosopher Derek Turner put it in seven slogans, “(1) Paleontology has more to contribute to biology than to geology; (2) Study fossils in bulk—individual specimens don’t tell you much about evolution; (3) Paleontology needs theories; (4) If you can’t experiment, then simulate; (5) don’t assume that the fossil record is incomplete; analyze the incompleteness; (6) resist reductionism; (7) don’t shy away from raising big questions about evolution” [19]. This does not mean, though, that all these features were literally invented by paleobiologists, but rather that Gould and colleagues put these elements at the center of their research programs.

Following these starting points, Gould asserted that paleontology was a legitimate biological discipline able to uncover patterns and mechanisms which could be found at the second and tier tiers of time [20]. For instance, at the second tier, Gould individuated phenomena which occur primary within a punctuated equilibrium-pattern (as he had formulated years earlier together with Niles Eldredge [21]), whereas the third tier is dominated by mass extinction phenomena. These patterns and mechanisms complete, expand, and in part revise the neo-Darwinian picture of evolution.

Nine months after his article "The Paradox of the first Tier" was submitted to *Paleobiology*, Gould wrote to the journal’s editor, Jack Sepkoski, explaining the necessity of an interaction between different and autonomous layers of the evolutionary theory:

Hierarchy, as here discussed in its genealogical context, is an ‘internalistic’ theory about evolution dynamics. And we need to formulate it properly if we are to tackle this internal dynamic with the other great mover of life’s patterns—the externals of geological history especially mass extinctions, that so impact life’s history…in other words, all the data that you and your colleagues are treating in such new and exciting ways. Hierarchy confronts the geological dynamic, and we will not get it right until we reformulate both sides. Gould to Sepkoski, 13 August 1985 in [5].

In this letter, Gould clearly affirmed the importance of reformulating the hierarchical model of evolution in the light of the momentous and exciting research techniques used in the investigation of mass extinctions. The reformulation Gould had in mind, however, concerned the entire paleontological discipline. He suggested a “nomothetic and idiographic” approach to the fossil record based upon David Raup and Sepkoski’s studies on the structure of the mass extinction [22,23,24].

The Kantian philosopher Wilhelm Windelband (1848–1915) coined the nomothetic vs. idiographic distinction. On May 4, 1894, he gave his rectoral address on the methodological differences between History and Natural Science at the Kaiser-Wilhelms-Universität Strasburg. During that address, he distinguished historical from natural sciences by focusing on the “formal character of their cognitive goals” [25]. Nomothetic disciplines aimed at formulating general laws and general judgments, while the ideographic sciences merely collected historical facts. The former were sciences of laws, the latter sciences of events: “the former teach what always is, the latter what once was” [25]. This distinction was not about the contents of the two sciences; but rather it was about how scientists produce knowledge. That means that the main difference between nomothetic and ideographical disciplines was methodological. With his programmatic statements expressed publicly in a paper, Gould intended to bridge the methodological gap between the natural and bio-historical sciences [20].

To accomplish the reformulation, Gould promoted a methodological synthesis. He clearly suggested this in commenting on Sepkoski’s study on Phanerozoic diversity [26,27]. Gould affirmed that, “Here we see an interesting and fruitful interaction of nomothetics and ideographics. The form of the model remains nomothetic—the ‘‘real’’ pattern arises as an interaction between two general curves of the same form, but with different parameters. Ideographic factors determine the parameters and then enter as boundary conditions into a nomothetic model” [20].

The ideographic factors mentioned by Gould derive from Sepkoski’s famous *Compendium*. It gave the required data a mathematical treatment of data. This in turn made visible what is invisible: the structure and development of the Phanerozoic diversity [28,29].

Hence, the contrast between paleontology and paleobiology was mainly based on disciplinary and methodological issues. Gould and colleagues first created a disciplinary space in which to insert their research agenda (they named their approach “paleobiology”). Successively, they sought to a possible methodological and disciplinary synthesis, in effect avoiding possible dichotomies. Famously and quoting German philosopher Immanuel Kant, Gould wrote “with all biology and no geology, paleontology is empty; but with geology alone, it is blind” [20]. Furthermore, by calling for a synthesis, another theoretical issue was tackled by twentieth-century paleobiology: the possible over- and under-determination of paleontological explanations.

## 3. Over- and Under-Determination

It is quite difficult to find well-preserved fossils that immediately resemble living organisms that can also be exhibited, used, and taken at face value. In fact, once an organism dies, it is subjected to the several taphonomic processes. These processes destroy and change the features of the original organism. For instance, it is rare to find fossils with their soft parts preserved. There are two ways to practically address this problem: (1) focus on structures that are more frequently preserved; (2) focus on exceptionally well-preserved sites. These provide more selective observations that can be essential, but have their limitations when studying rapid changes during evolution (I thank an anonymous reviewer who pointed this out to me. See [30,31])

The imperfect and incomplete nature of the paleontological record gave several paleontologists cause to reflect on the epistemic aspect of the fossil record. As a result, paleontologists came up with practices intended to overcome the incomplete nature of the records of the past. In addition to working with imperfect and incomplete data, paleontologists must face another difficulty: the so-called over and underdetermination issue.

As philosopher of science Carol E. Cleland has pointed out, historical sciences, such as paleontology, are subjected to the asymmetry of overdetermination [32,33]. They are in the same condition as the investigator who is trying to reconstruct what, exactly, shattered a window starting from the traces on the floor. Let us imagine that three different people throw different objects at the same window at the same time. In that case, “the breaking of the window is overdetermined by numerous sub collections of shards of glass lying on the kitchen floor. That overdetermination of earlier facts by later traces occurs whenever a window breaks” [34].

Following Turner, we can develop the thought experiment a bit further. “The owners of the house sweep up the shards, throw the baseball in the bin, and eventually repair the window. A few weeks later, the only traces of the event that remain are a few shards of glass under the refrigerator. The housecleaning and repair are examples of what Sober (1988, 3) calls *information-destroying processes*” [34].

Let us follow Turner again in this line of reasoning. Let us assume that a future investigator discovers glass shards on the kitchen floor. He will then ask what kind of shards they are—are they pieces of a glass, a window, a vase, etc.? “Even if the historical investigator recognizes the traces for what they are”, noted Turner, “rival hypotheses about earlier events and processes will often be underdetermined by the available traces. After studying the shards under the refrigerator, the historical investigator will be completely confused: The evidence does not permit her to discriminate at all between incompatible opposing hypotheses (window vs. wine glass, football vs. baseball, etc.) In other words, she confronts a local underdetermination problem” [34].

Therefore, the present event (a broken window, or, in paleobiology, a mass extinction) over- or under-determines its possible causes. Replaying the tape of time, we cannot be sure to identify the correct sequence of cause–effect, since there are many possible causal chains backwards from the local event.

Hence, first, the record of the past is always imperfect and incomplete, and second, we are not able to state whether our imperfect and incomplete data overdetermine or underdetermine the phenomena paleontologist would like to investigate.

Given these two issues, how can paleontologists bring out patterns and mechanisms that might expand evolutionary theory? Are these two issues not a death kiss for paleontology? And more broadly, what is paleontological business about? To answer these questions, I will briefly recall some research results Gould himself put in the middle of his agenda.

One main topic of twentieth-century paleobiology was the debate about the importance and dynamics of mass extinction [5,35]. One central research result was the famous paper (and graph) representing the periodicity in mass extinctions within geological time. It was used by Gould to indicate a classical phenomenon which happens on the third temporal tier. This representation has many interesting peculiarities, which are related to the notion of paleobiological data [16,29,36,37].

Due to Alvarez’s team discovery (1978) of the iridium anomaly in rocks formation at K–T boundary, mass extinction became the hot topic of paleontology in the 1980s. One of the main questions during those years was concerning the number and intensity of the mass extinctions.

This issue was resolved in two famous papers written by David Raup and Jack Sepkoski. Using Sepkoski’s database (1982), Raup and Sepkoski identified the number of mass extinctions and the periodicity of this phenomenon. Sepkoski and Raup plotted a huge number of fossil marine families against geological time and found that five mass extinctions clearly occurred in the history of the Earth as “statistically distinct from the background extinction levels”. These “five extinction events are seen as sharp drops in standing diversity”, and therefore they were easily detected. A mass extinction is thus an event in which a “large number of organisms have disappeared over relatively short time” [22,23].

This case study is emblematic since it shows the *highly integrative practice* used in twentieth-century paleobiology. As for the seminal investigation of morphogenesis conducted by David Raup and Adolf Seilacher during the 1960s and 1970s [11,38], also in this case paleobiologists sought to integrate data and technologies to produce possible coherent scenarios of the past. Since a direct access to the deep past is impossible, scientists technically recreate possible scenarios which tell the scientists that something happened in the past. Hence, to overcome the issue of over- and underdetermination, paleobiologists stretched and elaborated their data with the help of technology (such as computer, electron microscope, databases, etc.). The key methodological insight was indeed *the fruitful combination of science and technology*. This implied that phenomena such as mass-extinctions or a life-like display of extinct specimens in a museum’s hall depend both on the correct use of technological devices and on the interplay between these devices and theories. Hence, paleobiology can be seen as a phenomena-lead discipline. These “investigations are such because the relevance of evidence turns on relationships between phenomena, the hypotheses pertaining to them” [39] and, I would add, the technologies used to stage deep time. Shades of glass on the ground or fossil excavated in a particular region are evidence if these can be used into this integrative process [40].

## 4. Twenty-First-Century Paleontology: More Data, More Technology, and More Integration

What is left of the paleobiological research agenda? As I will detail in the conclusion, twentieth-century paleobiology shares with twenty-first-century paleontology the needs for integration and synthesis. In this section, I would like to briefly single out five promising research topics of current paleontology and connect them with twentieth-century paleobiology. I would like to characterize the research of twenty-first-century paleontology with a slogan: more data, more technology, and more integration.

The first topic I chose is about the emergence of paleocolor as a testable field of enquiry. To achieve this aim, it is important to have more clean data. In addition to the publication of studies on feather taphonomy, scientists are teaming up to use ion beam scanning electron microscopy to investigate the preserved melanin pigments. By integrating more data with more technologies, paleocolor can provided precious insights into the behavior and ecology of extinct organisms [34,41,42,43,44,45,46,47,48].

Second, as noted, one main issue paleontologists have to face is the lack of appropriate data. The clarification around the preservability of organic chemicals in fossils, and more broadly, the mechanisms behind taphonomy, is another key research program of twenty-first-century paleontology. Also in this case, the choice and use of appropriate technologies (such as mass spectrometry methodology, appropriate databases, etc.) is essential to obtain more data and thus pose new questions on phylogenetic hypotheses [49,50,51,52,53,54].

Third, another promising topic of twenty-first-century paleontology is given by the intersection between morphology, evolutionary theory, and various technologies. The intersection between robotics and morphology provides one compelling example. Scientists are working together with engineers to model and construct bio-inspired robots to understand evolution. One example above all (for another example published recently, see [55]) is given by research on the morphology of *Orobates pabsti*. This is a four-legged vertebrate organism that went extinct about 300 million years ago. The study of the morphology of this well-preserved specimen is very important because it could offer valuable insights into the evolution of terrestrial vertebrates. *Orobates* are an early evolution of the lineage that led to amniotes. These made the vertebrate transition to land by becoming independent of open water during the early stages of development. Thus, studying and understanding how these species were able to transition from water to land is essential to better understand one of the major transitions in vertebrate evolution.

To perform this research, scientists designed the OroBOT robot. It was designed to account for the locomotion dynamics of *Orobates*. The OroBot was built in collaboration with bioengineers at the École Polytechnique Fédérale de Lausanne (EPFL) in Lausanne. The spine of the OroBot was segmented into eight joints: two for the neck, four for the trunk, and two for the tail. The feet consisted of three passive and compliant joints. The designed parts of OroBOT were made of polyamide plastic material and created by selective laser sintering. Working in vivo with these robots, Nyakatura’s team was able to reach conclusions about the complex form-function that characterized the vertebrates’ transition to land [56,57,58,59].

The same logic is behind the creation of a chewing machine, an artificial mechanical chewing machine, used to study the micro-wear process and the possible correlation with diet and the rate of tooth wear. As the authors noted, “the aim was a simplified system that would allow changing various factors in mastication, for example different standardized feeds, to test whether expectations based on a simplistic interpretation of microscopic and macroscopic wear could be confirmed. The aim of this study was to produce microwear features seen in nature, i.e., pits and scratches, and to quantify macroscopic wear with real teeth and diets, in order to achieve a detailed picture of the wear process and its components” [60]. As in the case of the OroBot, the use of technology and various machines here also brings the investigation of the past (micro-wear process) close to engineering [59,61].

This synergy between technology and paleontological research has also taken different forms in the present day. Among them, one is virtual paleontology [62,63,64,65,66,67]. This leads to a second digital revolution in paleontology. This second digital revolution is signed by the passage from bio-robotics, or nature-inspired robotics, to robotics-inspired biology. This transition implies bridging of the gap between technology and nature. Form changes should now be studied through in vivo investigations (such as the classical anatomical dissection), in silico (as, for example, through CT scanners or computer simulations), and eventually again in a hybrid and highly integrated in vivo–silico–robotic environment through bio-robotics [61]. The full integration of these methodological levels would help illustrate the structural interplay of elements that characterizes form change.

The fourth hot topic of twenty-first-century paleontology is deeply rooted in the twentieth-century paleobiology. As noted in the introduction, paleobiology was launched to reorient the paleontological agenda toward broader evolutionary problems. This was the main reason for the paleobiological revolution of the 1960s and 1970s—think of Eldredge and Gould’s call for punctuated equilibrium. The same search for new evolutionary mechanisms pervaded paleobiological research during the first encounters with the emerging evo-devo community. Or rather, the evolution of evo-devo was shaped by paleobiological questions from the beginning, and vice versa. In fact, at the 1981 Dahlem conference on “Evolution and Development”, which will be considered as the grounding meeting of evolutionary developmental biology as an autonomous evolutionary discipline, biologists and paleontologists discussed together the relationship between evolution and development. For instance, in the working group on “The Role of Development in Macroevolutionary Change” biologists, such as Jim Murray, Pere Alberch, Brian Goodwin, Gunther Wagner, Tony Hoffman, and David Wake, defined with paleontologists Stephen Gould, Adolf Seilacher, and David Raup the role of constrains in evolution [11,68,69,70].

The same line of continuity and interaction denotes current paleontology, which even formalizes the paleontological contribution to evo-devo as Paleo-Evo-Devo. Therefore, the paleontology of the twenty-first century is also characterized by the search for a strong integration in this fourth case [71,72,73,74,75,76,77,78,79].

The fifth topic I have singled out is also anchored in the twentieth-century paleobiological agenda: the discussion of abiotic and biotic factors in evolution. Recall that I opened this paper with a quote from Maynard Smith. He welcomed the return of paleontology to the high table as a result of Raup and Sepkoski’s findings on mass extinction and the consequent focus on biotic elements in evolution. This research, in turn, had emerged from the study of the dynamics of Phanerozoic marine paleodiversity (and the proposed model analyses for studying diversity) and the Red Queen model proposed by Leigh Van Valen [5].

Twenty-first-century paleontology is capitalizing on this line of research. At the same time, paleontologists are trying to synthetize biotic and abiotic factors. For instance, Mike Benton notes, “The realization that the Red Queen and Court Jester models may be scale-dependent, and that evolution may be pluralistic, opens opportunities for dialog” [80]. He goes on asserting, “methods are shared by paleontologists and neontologists, and this allows direct communication on the patterns and processes of macroevolution” [80]. Fortelius and colleagues are also proposing “a tentative synthesis, characterized by interdependence between physical forcing and biotic interactions” [81].

Along these lines, paleontologist Tyler Faith and his colleagues emphasized the key methodological elements of current hominin evolution. First, paleoanthropology, as paleontology, is all about data and time scales (or as Gould put it tiers): “There is no universally ‘correct’ scale of observation, but to address questions linking ecology and evolution, the scales of the processes of interest must align with the scales of the available data” [82]. Second, paleoanthropology is desperately in need of more theory (recall Turner’s third point for describing paleobiology), or rather of a new hypothetic-deductive methodology [83]: “Incorporating a stronger theoretical framework into the agenda of hominin paleoecology will allow researchers to reverse the typical direction of inference (i.e., from data to hypothesis) by generating theoretically informed predictions that are tested with the data, and then determining if a hypothesis should be modified or rejected” [82]. By granting a new methodology, a new balance may emerge “between inferring evolutionary narratives from the data and testing process-based hypotheses using those data” [82]. Hence, again, twenty-first-century paleo-research is about more data, more technology, and more integration.

## 5. Conclusions: A Plea for a New Synthesis

What do the five topics just discussed tell us about the elements of continuity between twentieth-century paleobiology and twenty-first-century paleontology? First, both enterprises called paleontologists’ attention to a *cooperative effort* to understand the (deep) past. As paleontological data are always imperfect and incomplete, paleontologists should omnivorously assimilate every method to successfully and opportunistically work on and with deep time [40,84,85]. Molecular approaches to the deep past are therefore supplementary not antagonistic to classical paleontological analyses of forms [86]. Sometimes, they allow paleontologists to see things better or, at least, in a different fine grade (e.g., [87]). How, however, can paleontologists confidently use data, names, technology, and knowledge outside their field of application if they do not know the correct boundaries of application of such tools in their own? Critical integration of different methods can help mediate and hopefully overcome technical issues. This should be paired with inter- and multidisciplinary programs to enable students and scholars to fruitfully learn and apply different methods—that was one of insights of the paleobiological revolution of the 1960s and 1970s.

Indeed, this implies a return to the sprit embodied by the paleobiological revolution of the mid twentieth-century. At the end, what Gould and colleagues were asking for was a genuine synthesis of knowledge. They asked for blurring the disciplinary borders between natural and historical sciences as well as between science and technology. This spirit should be put at the center of twenty-first-century paleontology – this agenda is also perused by other technoscientific disciplines such as biorobotics, synthetic biology, nano(bio)technology, etc. See, for instance, [59,61,88,89,90,91]. This enterprise should be characterized by a cooperative model of knowledge production. Instead of supporting a never-ending disciplinary struggle to define what paleontology is (or is not), to draw a sharp line between paleontology and neontology, or between morphological and molecular phylogenetics, scholars should work together and ideally share their data and method to address new challenges, as has happened during periods of major theoretical transitions in the history of paleontology [5,29,92]. The results provided by Perri et al. [93] are a clear example of the continuum of approaches that characterizes paleontology and biology.

A future task for philosophers and historians of paleontology would be to understand the various practices and reasons for the paleontological plea for synthesis and integration in the last century. In other words, the analysis should focus on what (and why) paleontologists sought (and are seeking) collaboration with neontologists and promote the circulation of knowledge and technologies [94] (an important starting point would be the analysis of how paleontologists and neontologists are publishing together or taking part in joint conference. I thank one referee for this point. See, for instance, the papers gathered in the special issue “Crossing the Palaeontological-Ecological Gap” published in *Methods in Ecology and Evolution* 2016, 7. Furthermore, it is perhaps worth noting that Wolfgang Kiessling was the first paleontologist to play a major role in the Intergovernmental Panel on Climate Change (IPCC) report on climate change, working on ”Climate Change 2021: Impacts, Adaptation and Vulnerability“, thus bridging the gap between the two communities - I thank one referee for this. See also [35,95,96,97]). 

This point will make visible the role of the so-called invisible technicians, i.e., all those who work (silently and without much recognition) on the production of paleontological knowledge. In addition to the flood of data and the transformation of the paleontologist into a data scientist (and vice versa), *field and preparatory work needs to be recognized as it remains at the heart of paleontology today* [98,99,100]. Today, more than ever, the classic metaphor of the earth as an archive of knowledge and data is still valid. In fact, the greatest amount of data is still buried in the field. On the importance of field work for paleontology [101,102,103,104,105,106]. This focus will create an awareness of the new social hierarchies in twenty-first century paleontology that result from the massive use of technology.

Hence, and to conclude, if constructively interpreted and read through the recent history of paleontology, the five topics singled out may provide some potentialities for a new synthesis between genetic and morphological approaches to the evolution of forms in the attempt to overcome the limits, issues, and problems of phylogenetic reconstructions. The major implication would be a broader reflection on what paleontology might become in light of recent technical and molecular revolutions. This would imply a second paleobiological revolution. In fact, as David Sepkoski put it, “the paleobiological “revolution” was more like a political contest in which one group perceives itself to be disenfranchised and agitates for greater representation in government than a contest of lofty ideas. The 1970s was a period of revolution in paleontology because paleobiologists saw themselves, and described what they were doing, as revolutionary” [5]. Echoing the paleobiological revolution of the 1960s and 1970s, a twenty-first-century paleobiological revolution would enable paleontologists to gain strong political representation and to argue with a decisive voice at “the high table” on topics such as the extended evolutionary synthesis, the Anthropocene, conservation of Earth’s environment, and global climate change.

## Data Availability

Not applicable.
